# Frequency of hyperkalemia during optimization of guideline-directed medical therapy in ambulatory patients with HFrEF

**DOI:** 10.3389/fcvm.2025.1562647

**Published:** 2025-05-09

**Authors:** Genaro H. Mendoza-Zavala, Gibran Reynoso-Hernandez, Edith L. Posada-Martinez, Miguel Sandoval-Jimenez, Jairo I. A. Alejo-Arcos, Kassandra Rios-Felix, Eileen Amaro-Balderas, Marisol Gomez-Lopez, Sonia C. Juarez-Comboni, Luis F. Tejado-Gallegos, Emerson Joachin-Sanchez, Luis Olmos-Dominguez, Moises Aceves-Garcia, Marco J. Olalde-Roman, Marissa A. Silva-Garcia, Eduardo Almeida-Gutierrez, Cristina Revilla-Monsalve, Adolfo Chavez-Mendoza, Jose A. Cigarroa-Lopez, Jonathan S. Chávez-Iñiguez, Jose A. Magaña-Serrano, Juan B. Ivey-Miranda

**Affiliations:** ^1^ Department of Heart Failure, Hospital de Cardiología, Instituto Mexicano del Seguro Social, Mexico City, Mexico; ^2^ Department of Echocardiography, Instituto Nacional de Cardiologia Ignacio Chavez, Mexico City, Mexico; ^3^ AstraZeneca, Mexico City, Mexico; ^4^ Department of Education and Research, Hospital de Cardiología, Instituto Mexicano del Seguro Social, Mexico City, Mexico; ^5^ Unidad de Investigacion Medica en Enfermedades Metabolicas, Hospital de Cardiología, Instituto Mexicano del Seguro Social, Mexico City, Mexico; ^6^ Department of Nephrology, Hospital Civil de Guadalajara Fray Antonio Alcalde, Guadalajara, Mexico

**Keywords:** heart failure, GDMT, hyperkalemia, spironolactone, potassium binders

## Abstract

**Background:**

The frequency of hyperkalemia in patients with heart failure with reduced ejection fraction (HFrEF) receiving a high percentage of quadruple guideline-directed medical therapy (GDMT) has not been described extensively. The consequences of hyperkalemia on modifications in GDMT have not been fully addressed in patients receiving quadruple therapy.

**Methods:**

This was a retrospective cohort study of outpatients with HFrEF treated at a specialized heart failure clinic. A case-by-case retrospective review of patients fulfilling the selection criteria was conducted by dedicated personnel. The main exposure was the occurrence of hyperkalemia at any visit, and the primary outcome was the modification in GDMT following hyperkalemia.

**Results:**

We included 1,279 medical encounters from 500 unique patients. Over a mean follow-up of 11 ± 7 months (2.6 ± 0.9 visits), the proportion of patients receiving angiotensin-converting enzyme inhibitor/angiotensin receptor blocker/angiotensin receptor-neprilysin inhibitor, beta-blockers, spironolactone, sodium-glucose co-transporter 2 inhibitors (SGLT2is), and quadruple therapy increased to 98.6%, 99.0%, 97.4%, 93%, and 89.6%, respectively (*P* < 0.001 compared to baseline). The proportion of hyperkalemia during follow-up, defined as serum potassium >5.0,>5.5, and >6.0 mmol/L at any visit, was 44.4%, 13.0%, and 4.0%, respectively. In multivariable analysis, estimated glomerular filtration rate was the only independent predictor of hyperkalemia across all cutoff values (*P* < 0.001 for all). Serum potassium was associated with greater odds of mineralocorticoid receptor antagonist (MRA) discontinuation in a non-linear fashion, with an increased risk starting at >5.0 mmol/L (*P* < 0.001). Initiation of SGLT2is was not associated with lower odds of developing hyperkalemia at subsequent visits (*P* > 0.20 for all cutoff values).

**Conclusions:**

Hyperkalemia >5.0 mmol/L is highly prevalent in patients with HFrEF receiving quadruple GDMT. Even with mild hyperkalemia, discontinuation of MRAs remains the primary strategy for managing this complication.

## Introduction

Patients with heart failure (HF) are at increased risk of hospitalization and death (1, 2). However, in HF with reduced ejection fraction (HFrEF), guideline-directed medical therapy (GDMT) has significantly improved outcomes, leading to a 64% reduction in the composite of cardiovascular mortality and heart failure hospitalizations (3). Unfortunately, data from large international registries revealed that optimal GDMT remains insufficiently achieved, with the mineralocorticoid receptor antagonist (MRA) class being the less common prescribed drug (4–6). The cause for this low GDMT is multifactorial, but concerns about hyperkalemia are a well-recognized barrier (7–9).

Hyperkalemia is a well-known marker of severity and poor prognosis in patients with HFrEF (7–9). In large registries, the prevalence of hyperkalemia in HFrEF reaches up to 24.4% (10), a complication that can be explained by coexisting comorbidities, such as chronic kidney disease or diabetes (11), which can occur in up to 60% of people with HFrEF (12); additionally, therapies, such as renin-angiotensin-aldosterone system inhibitors or beta-blockers (BBs), can contribute to the risk of HFrEF (13). Notwithstanding, the real occurrence of HFrEF in patients receiving quadruple GDMT remains unknown. Interestingly, the use of sodium-glucose co-transporter 2 inhibitors (SGLT2is) has been associated with a lower risk of developing hyperkalemia (14, 15), but their effect on established hyperkalemia remains unknown. Likewise, data regarding changes in GDMT following the detection of hyperkalemia are required in patients receiving quadruple GDMT.

Therefore, in the present study, we (1) evaluated the frequency of hyperkalemia transversally and longitudinally in a contemporary cohort of patients with HFrEF during the optimization of GDMT to achieve quadruple therapy, (2) assessed the impact of hyperkalemia on GDMT, and (3) described the effect of modifying GDMT on subsequent serum potassium levels.

## Methods

This study is a retrospective analysis of a cohort of patients with HFrEF treated at a specialized heart failure clinic (Hospital de Cardiologia in Mexico City). Demographic information, comorbidities, medication use, and laboratory data were obtained from electronic medical records (EMRs). An MD with specialized training in HFrEF conducted a case-by-case retrospective review of all patients who met the selection criteria. We included ambulatory patients who were seen as part of their regular follow-up visits starting 1 January 2020. Inclusion criteria were diagnosis of HFrEF for at least 3 months, age ≥18 years, no readmissions within the last 30 days, an echocardiogram performed within the last year showing an left ventricular ejection fraction (LVEF) ≤40%, availability of data regarding serum potassium and creatinine levels at baseline and at least one follow-up visit, and complete data regarding medical treatment. Exclusion criteria included patients with hyperaldosteronism, an estimated glomerular filtration rate (eGFR) of <15 ml/min/1.73 m^2^ at the first medical encounter, or those undergoing dialysis. Chronic kidney disease was defined as an eGFR of <60 ml/min/1.73 m^2^ for more than 3 months, as estimated by the Chronic Kidney Disease Epidemiology Collaboration (CKD-EPI) equation and in accordance with Kidney Disease: Improving Global Outcomes (KDIGO) guidelines (16). To reduce the risk of selection bias, all consecutive EMRs from the date described above were screened to be included in this analysis, and only EMRs from patients who met all the inclusion criteria and none of the exclusion criteria were analyzed. To reduce the risk of information bias, we included only patients who continued follow-up at our clinic. In addition, to reduce the risk of confounding bias, the MD collected specific data on variables that could potentially confound the association between serum potassium levels and modifications in GDMT.

The main exposure was the occurrence of hyperkalemia at any visit, and the primary outcome was the modification in GDMT following hyperkalemia. Thus, the baseline characteristics of patients were compared based on the presence of hyperkalemia at any time during follow-up, as presented in Table 1. In addition, the baseline characteristics of patients are also compared by the presence of hyperkalemia at baseline in the Supplementary Table. The study was approved by the local IRB at the Hospital de Cardiologia, and the requirement of informed consent was waived, as this was a retrospective review of EMRs.

**Table 1:** Baseline characteristics of patients based on the presence of mild hyperkalemia at least once during follow-up.

Characteristics	Total cohort *N* = 500	Serum K < 5.0 mmol/L *N* = 278 (55.6%)	Serum K ≥ 5.0 mmol/L *N* = 222 (44.4%)	*P*-value
Age (years)	58 ± 13	56 ± 13	61 ± 11	<0.001
Male	352 (70%)	195 (70%)	157 (71%)	0.89
BMI (kg/m^2^)	26.8 (24.5–29.7)	27.2 (24.5–30.1)	26.5 (24.5–28.8)	0.06
LVEF (%)	27 ± 8	27 ± 8	28 ± 8	0.49
Comorbid conditions				
Hypertension	204 (41%)	108 (39%)	96 (43%)	0.32
Diabetes mellitus	225 (45%)	115 (41%)	110 (50%)	0.07
Ischemic heart disease	285 (57%)	143 (51%)	142 (64%)	0.005
Chronic kidney disease	145 (29.0%)	61 (21.9%)	84 (37.8%)	<0.001
Vital signs				
Systolic blood pressure (mmHg)	111 (100–125)	112 (100–126)	110 (100–122)	0.43
Heart rate (bpm)	74 (66–85)	75 (68–86)	74 (65–85)	0.07
Medications at baseline				
ACEi, ARB, or ARNI	471 (94%)	262 (94%)	209 (94%)	0.96
Beta-blocker	470 (94%)	257 (92%)	213 (96%)	0.10
Spironolactone	449 (90%)	250 (90%)	199 (90%)	0.92
SGLT2i	276 (55%)	156 (56%)	120 (54%)	0.65
Laboratory values^a^				
Serum sodium (mmol/L)	140 (138–142)	140 (138–142)	140 (138–142)	0.57
Serum potassium (mmol/L)	4.5 (4.2–5.0)	4.4 (4.1–4.6)	5 (4.5–5.3)	<0.001
Serum chloride (mmol/L)	104 ± 4	104 ± 4	104 ± 4	0.45
Serum creatinine (mg/dl)	1.1 (0.9–1.3)	1.1 (0.9–1.2)	1.2 (1–1.4)	<0.001
Urea (mg/dl)	44 (34–58)	41.6 (31.8–53.0)	48.0 (38.0–66.0)	<0.001
eGFR (ml/min/1.73 m^2^)	72 ± 24	77 ± 24	66 ± 23	<0.001
NT-pro-B-type natriuretic peptide (pg/ml)	1,496 (442–3,460)	1,307 (409–3,232)	2,196 (518–4,549)	0.21

BMI, body mass index; LVEF, left ventricular ejection fraction; ACEi, angiotensin-converting enzyme inhibitor; ARB, angiotensin receptor blocker; ARNI, angiotensin receptor-neprilysin inhibitor; eGFR, estimated glomerular filtration rate.

Values are presented as *n* (%) or mean ± standard deviation unless specified.

^a^ Laboratory levels are shown at the first medical encounter.

This study was conducted according to the Strengthening the Reporting of Observational Studies in Epidemiology guidelines and the REporting of Studies Conducted using the Observational Routinely Collected Health Data statement (17, 18). We adhered to the principles outlined in the Declaration of Helsinki for medical research involving human participants and ensured ethical standards and methodological transparency.

### Definition of hyperkalemia and modifications in GDMT

Hyperkalemia was defined as mild, moderate, or severe if the serum potassium level was >5.0, >5.5, or >6.0 mmol/L, respectively (19). The MD, doing the case-by-case retrospective review, specifically collected if there were modifications in GDMT following the detection of hyperkalemia. Participants were followed until the last follow-up visit, and the database was locked in July 2024.

### Statistical analysis

Continuous data that were approximately normally distributed are presented as mean ± standard deviation, while data with skewed distribution are described as median (quartile 1–quartile 3). Categorical data are reported as frequency (%). Comparisons of continuous data were performed using Student's *t*-test or the Mann–Whitney *U*-test, as appropriate. Categorical data were compared using the chi-squared test. Paired categorical data were compared using the Wilcoxon matched pairs signed-rank test. Skewed variables were log-transformed. Predictors of hyperkalemia at the patient level (no repeated measures) were analyzed by multivariable logistic regression models. Covariables included were age, sex, body mass index, hypertension, diabetes, coronary artery disease, use of angiotensin-converting enzyme inhibitors/angiotensin receptor blockers/angiotensin receptor-neprilysin inhibitors (ACEis/ARBs/ARNIs), beta-blockers, MRAs, SGLT2is, and the eGFR. Stepwise backward elimination was used, and only variables with *P* < 0.05 were retained as independent predictors. The association between hyperkalemia and eGFR and the association between discontinuation of any GDMT and serum potassium levels were analyzed using repeated measures with multilevel mixed-effects logistic regression to account for the absence of independence of observations. The mixed models were two-level models with random intercepts by a variable identifying each patient. eGFR and serum potassium levels were modeled using restricted cubic splines with three knots placed at recommended percentiles and plotted to capture their relationship with the odds of hyperkalemia or odds of GDMT discontinuation, respectively. The odds of MRA discontinuation were also assessed using multilevel mixed-effects logistic regression adjusting for age, sex, diabetes mellitus, hypertension, functional class, systolic blood pressure, eGFR, and serum sodium. Statistical significance was defined as two-tailed *P* < 0.05. All statistical analyses were performed using Stata SE, version 18.0 (StataCorp, College Station, TX, USA).

## Results

### Baseline characteristics

We included a total of 1,279 medical encounters from 500 unique patients. Table 1 describes the baseline characteristics of the overall cohort and those stratified by the occurrence of hyperkalemia >5.0 mmol/L in at least one visit. Patients with hyperkalemia were more likely to be older and to have ischemic heart disease, lower eGFR, chronic kidney disease, and higher serum urea. Results were similar when comparing patients with hyperkalemia >5.0 mmol/L at baseline (older, lower eGFR, chronic kidney disease, and higher serum urea).

**Figure 1:**
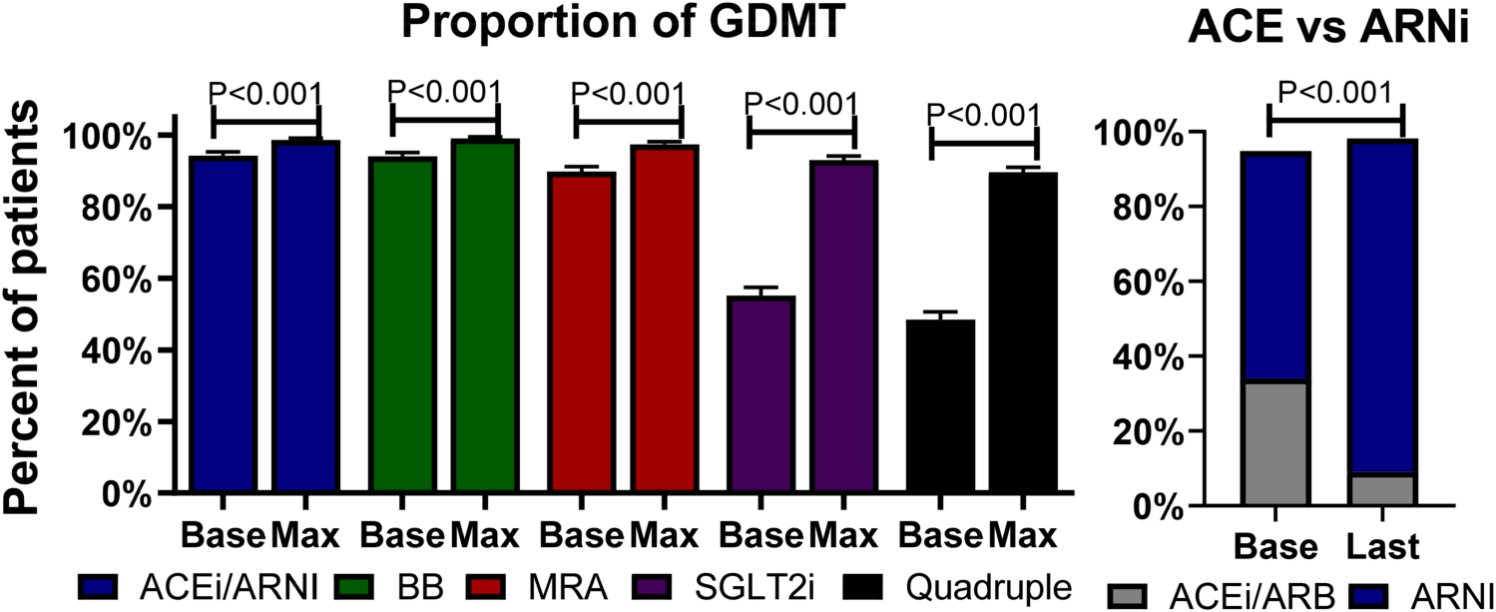
Proportion of GDMT. Left panel: bars describe the percentages and standard errors of the prescription of each category of GDMT at baseline (Base) and the maximum (Max) percentage at follow-up. Right panel: Bars describe the percentage of ACEis/ARBs at baseline and the last follow-up visit. ACEi, angiotensin-converting enzyme inhibitor; ARB, angiotensin receptor blocker; ARNI, angiotensin receptor-neprilysin inhibitor; BB, beta-blocker; GDMT, guideline-directed medical therapy; MRA, mineralocorticoid receptor antagonist; SGLT2i, sodium-glucose co-transporter 2 inhibitor.

At the first medical encounter (baseline visit), the proportion of patients receiving ACEis/ARBs/ARNIs, beta-blockers, MRAs, and SGLT2is was 94.2%, 94.0%, 89.8%, and 55.2%, respectively, with 48.4% receiving quadruple therapy. During a mean follow-up of 11 ± 7 months corresponding to 2.6 ± 0.9 visits, the proportion of prescription of ACEis/ARBs/ARNIs, beta-blockers, spironolactone, and SGLT2is increased to a maximum of 98.6%, 99%, 97.4%, and 93%, respectively (*P* < 0.001 for all), leading to an 89.6% of patients on quadruple therapy (*P* < 0.001); see Figure 1, left panel. Importantly, the proportion of ACEis/ARBs decreased, while the proportion of ARNIs increased (*P* < 0.001 for both); see Figure 1, right panel.

### Frequency and predictors of hyperkalemia

**Figure 2:**
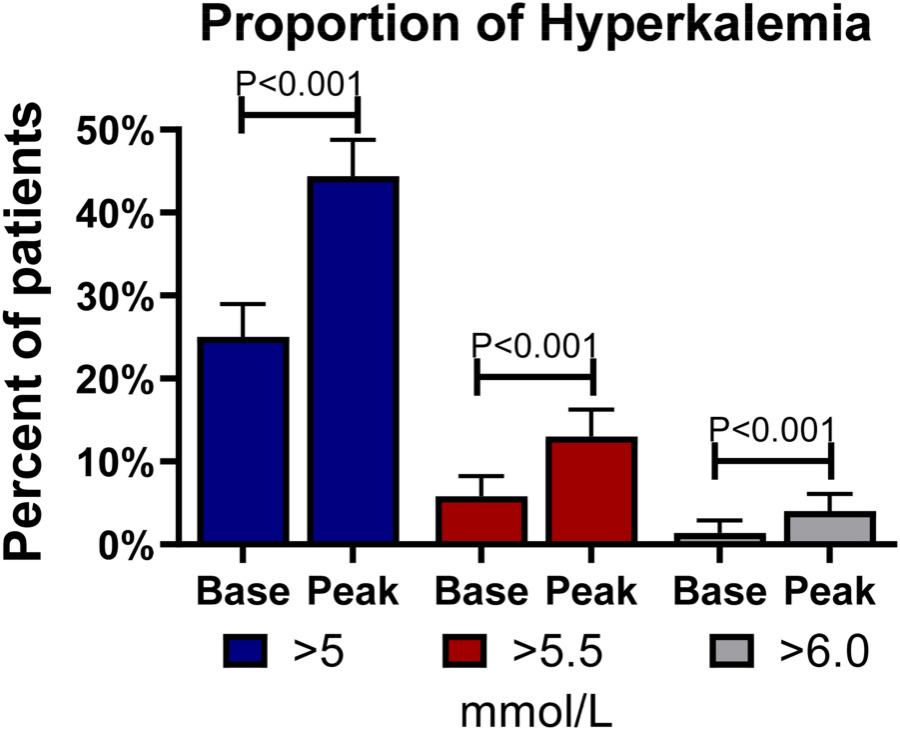
Frequency of hyperkalemia at baseline and follow-up. Bars describe the percentages and standard errors of hyperkalemia at different cutoffs of serum potassium at baseline (Base) and any follow-up visit (Peak).

**Figure 3:**
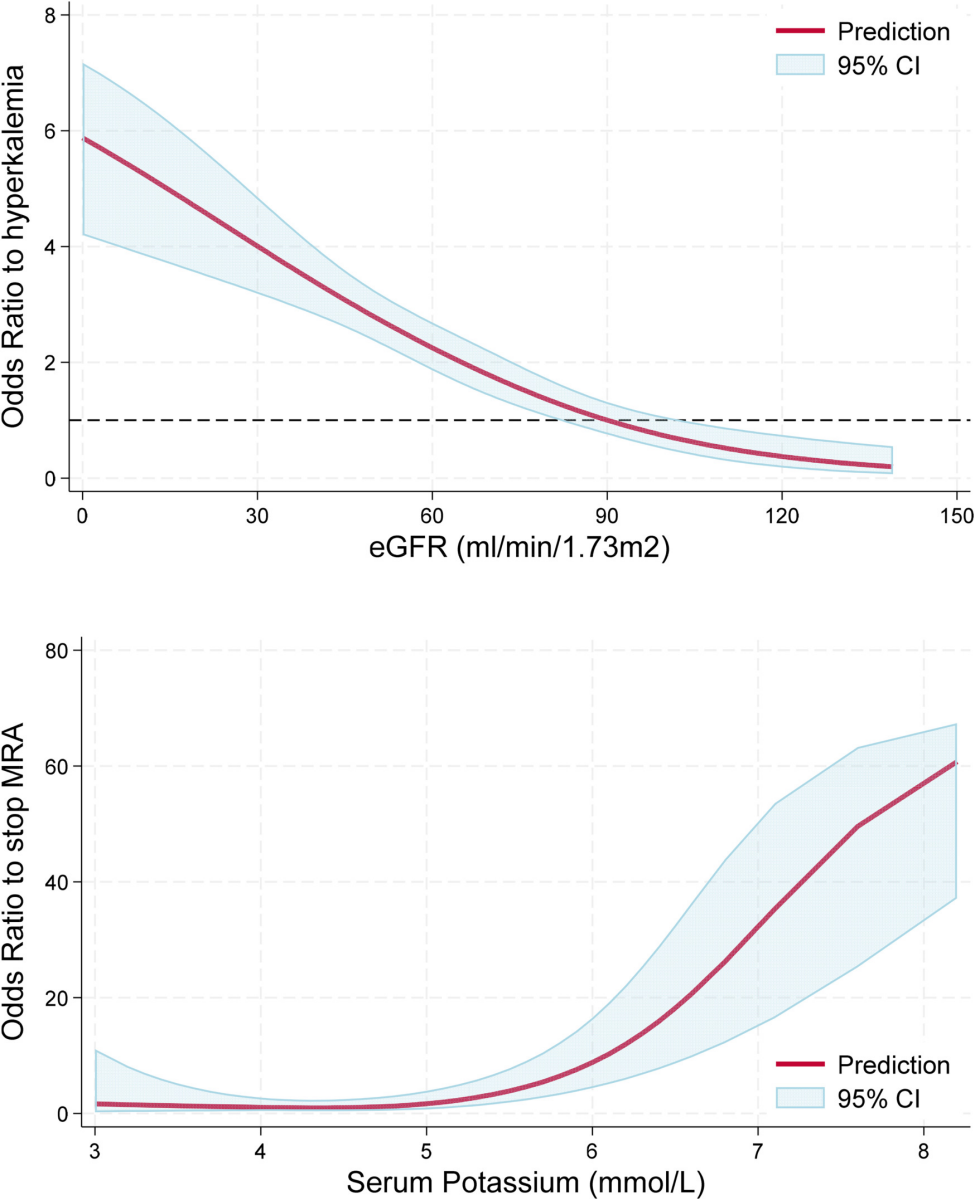
Association between hyperkalemia and eGFR, and serum K and odds of MRA discontinuation. Top panel: Lower eGFR was associated with greater odds (*P* < 0.001) of having hyperkalemia >5.0 mmol/L. The red line represents the odds for hyperkalemia as a function of eGFR relative to a reference value of 90 ml/min/1.73 m^2^. Bottom panel: Higher serum potassium levels were associated with greater odds of MRA discontinuation (*P* < 0.001) with a non-linear association (*P* for non-linearity = 0.024) characterized by increased risk with serum potassium levels >5.0 mmol/L. The red line represents the odds of MRA discontinuation as a function of serum potassium relative to a reference value of 4.5 mmol/L.

The proportion of hyperkalemia >5.0, >5.5, and >6.0 mmol/L at the first medical encounter was 25.0%, 5.8%, and 1.4%, respectively. However, the proportion of hyperkalemia at the same cutoff values at any visit during follow-up was 44.4%, 13.0%, and 4.0%, respectively (*P* < 0.001 for all); see Figure 2. In the multivariable analysis, independent predictors of hyperkalemia >5.0 mmol/L included age [OR: 1.18, 95% confidence interval (CI): 1.01–1.39, *P* = 0.047, per 10 years older] and eGFR (OR: 0.83, 95% CI: 0.76–0.91, *P* < 0.001, per 10 ml/min/1.73 m^2^ higher). Figure 3 (top panel) describes the odds of having hyperkalemia >5.0 mmol/L by the eGFR centered at 90 ml/min/1.73 m^2^. In the multivariable analysis, eGFR remained the only independent predictor of hyperkalemia >5.5 mmol/L (OR: 0.71, 95% CI: 0.63–0.80, *P* < 0.001, per 10 ml/min/1.73 m^2^ higher) or >6.0 mmol/L (OR: 0.69, 95% CI: 0.57–0.83, *P* < 0.001, per 10 ml/min/1.73 m^2^ higher).

### Effect of hyperkalemia on GDMT

**Figure 4:**
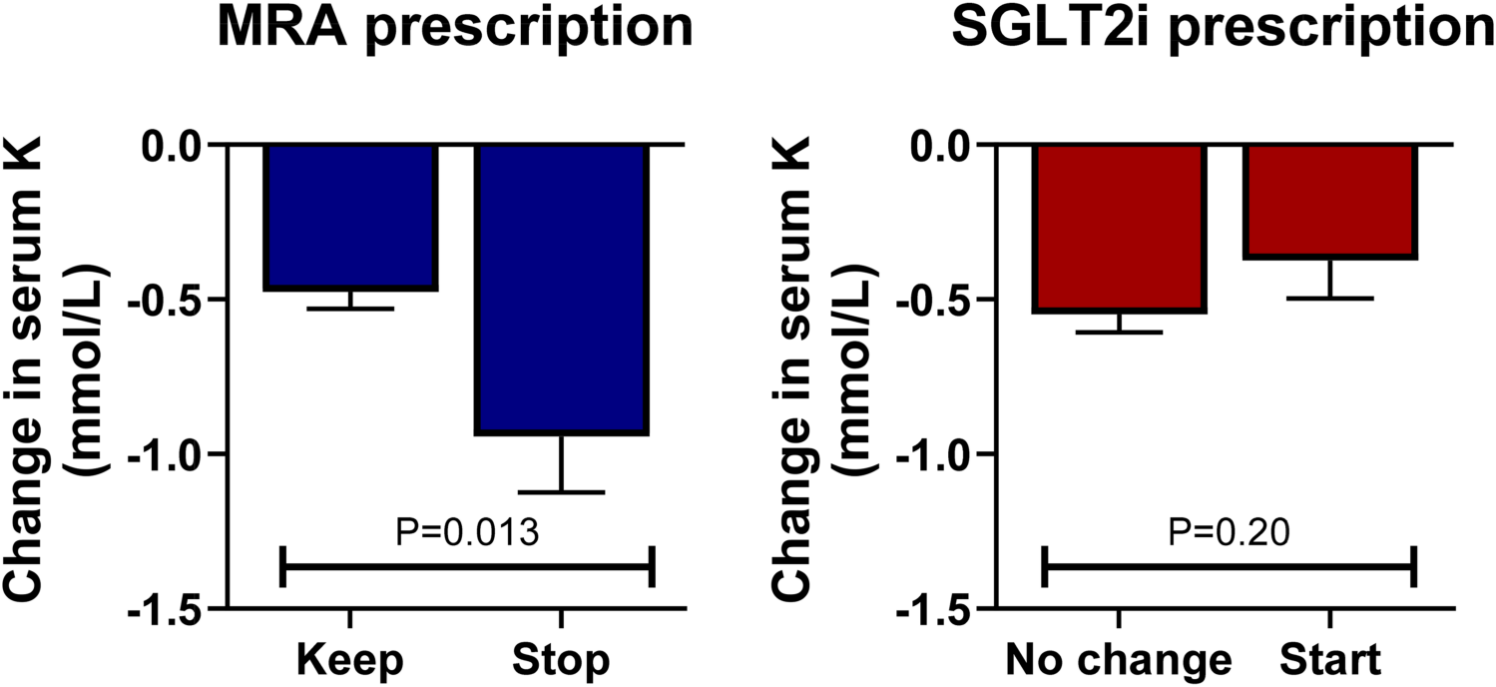
Changes in serum K levels by MRA and SGLT2i prescription in patients with serum K > 5.0 mmol/L. In patients with hyperkalemia (serum potassium >5.0 mmol/L), discontinuing the MRA antagonist was associated with a greater decrease in serum potassium compared with continuing this drug at the subsequent visit (left panel). The initiation of an SGLT2i in this setting was not statistically associated with a decrease in serum potassium (right panel).

At follow-up, a total of 313 events of hyperkalemia >5.0 mmol/L were observed, representing 24.5% of the 1,279 medical encounters. Serum potassium levels were associated with greater odds of MRA discontinuation in a non-linear fashion, as observed in Figure 3, bottom panel (*P* < 0.001), but they were not statistically associated with the discontinuation of ACEis/ARBs/ARNIs or beta-blockers (*P* > 0.60 for both). When repeating this analysis in multivariable logistic regression adjusting for age, sex, eGFR, and the use of ACEs/ARBs/ARNIs, beta-blockers, and SGLT2, serum potassium levels remained independently associated with greater odds of MRA discontinuation (*P* < 0.001). Starting an SGLT2i was not associated with lower odds of developing hyperkalemia at the subsequent visit (*P* > 0.20 for all cutoff values). Importantly, in patients with hyperkalemia >5.0 mmol/L, we observed a decrease in serum potassium of 0.47 mmol/L (95% CI 0.37, 0.58 mmol/L, *P* < 0.001) at the subsequent visit without any documented modification in GDMT; however, the decrease in serum potassium doubled when the MRA was discontinued (0.94 mmol/L, 95% CI: 0.59, 1.30 mmol/L, *P* < 0.001), which was a statistically significant larger effect by 0.47 mmol/L (95% CI: 0.10, 0.84 mmol/L, *P* = 0.013); see Figure 4, left panel. In this subgroup of patients with mild hyperkalemia, the initiation of an SGLT2i was not statistically associated with a decrease in serum potassium (*P* = 0.20); see Figure 4, right panel.

## Discussion

In this study of ambulatory HFrEF patients with ∼90% receiving quadruple GDMT, we observed that hyperkalemia was frequent, with 44% experiencing mild hyperkalemia and 13% moderate hyperkalemia (serum potassium ≥5.0 and ≥5.5 mmol/L, respectively) at least once during follow-up. Mild hyperkalemia was associated with more odds of MRA discontinuation, and this risk increased exponentially with increasing serum potassium levels.

While the prevalence of hyperkalemia has been reported in large registries, granular data on its frequency in cross-sectional and longitudinal analysis in real-world settings has not been extensively documented. Interestingly, in this study of patients with HFrEF, ∼90% were receiving quadruple therapy, and the proportion of mild hyperkalemia appeared to be somehow greater than in previous studies (8, 10). Whether this higher frequency of hyperkalemia is explained by the higher proportion of GDMT or influenced by other factors warrants further investigation. Despite the higher proportion of hyperkalemia, results were consistent with previous reports, confirming that eGFR is the most important predictor of hyperkalemia, irrespective of the cutoff value (8, 10, 11). This highlights the importance of monitoring serum potassium with closer follow-ups in high-risk populations, such as those with reduced eGFR, to enable timely treatment and, potentially, prevent its occurrence using therapeutic options that are now available (20, 21). Importantly, we found that hyperkalemia significantly impacted GDMT, with MRA discontinuation being the factor associated with its occurrence. While the internal consensus at our heart failure clinic recommends not discontinuing any GDMT unless serum K exceeds 5.5 mmol/L, we observed that the odds of MRA discontinuation start with mild hyperkalemia. We did not identify any other GDMT modifications related to hyperkalemia, nor did we find evidence that the addition of SGLT2 inhibitors was associated with lower odds of having hyperkalemia at the subsequent visit. While the discontinuation of MRAs may help lower serum potassium levels, it is known to potentially increase mortality (7, 22, 23). This is particularly relevant now that we have novel agents, such as sodium zirconium cyclosilicate and patiromer, which offer an excellent risk–benefit profile and tolerability and are cost-effective, with promising results in the management of hyperkalemia (21). In fact, guidelines and expert recommendations suggest using potassium-lowering agents when serum potassium exceeds 5.0 mmol/L (24, 25). Although most patients with hyperkalemia will have serum potassium levels between 5.0 and 5.5 mmol/L and may not require immediate initiation of these new therapies, physicians considering discontinuing or reducing GDMT should be encouraged to use these agents.

Study limitations: Although this cohort reflects a real-world population, we acknowledge that it is based on single-center data, which may differ from findings at other institutions. Another key limitation is the failure to capture other metrics relevant to hyperkalemia, such as albuminuria, glycemic control, urinary obstruction, or acid–base status. The average follow-up duration was 11 months; thus, these results might differ at longer follow-ups. In addition, the high rate of quadruple therapy utilization for HFrEF in our cohort may not fully represent the real-world patterns observed in other registries. The association between higher serum potassium levels and MRA discontinuation remained statistically significant in multivariable analysis; however, we could not account for other variables such as diet, breast pain, and other symptoms likely related to the decision to discontinue MRA. The main limitation of this study is that it lacks data regarding the association of high serum potassium levels and MRA discontinuation with clinically relevant outcomes such as mortality or heart failure readmission. However, the association between GDMT discontinuation and worse outcomes has been consistently reported in different studies involving patients with hyperkalemia. Thus, it is not very likely that such association would be significantly different in this cohort of patients with HFrEF and hyperkalemia. Notwithstanding, this issue still represents a significant limitation. Importantly, the lack of association between SGLT2i initiation and a reduction in serum potassium levels might be affected by selection bias, as this was not a randomized trial. In addition, several other factors, such as changes in diet and modifications in medications not captured in this study, might affect the observed results. Finally, another critical limitation is that no patients were prescribed potassium binders during follow-up, as these drugs are not yet available at our institution. However, it would have been very important to describe the role of these drugs in potentially reducing the discontinuation of MRAs or other drugs while maintaining serum potassium homeostasis in these patients.

In conclusion, we found that mild hyperkalemia affects 44% of patients with HFrEF receiving quadruple GDMT, with eGFR being the most important predictor. Even with mild hyperkalemia, discontinuation of MRAs remains the primary strategy for managing this complication, which significantly reduces serum potassium levels and likely reinforces the perpetuation of this conduct. The initiation of SGLT2is was not associated with a decrease in serum potassium levels in patients with detected hyperkalemia.

## Data Availability

The raw data supporting the conclusions of this article will be made available by the authors without undue reservation.
