# Supplementary material for: Frequency of hyperkalemia during optimization of guideline-directed medical therapy in ambulatory patients with HFrEF
Source: Front Cardiovasc Med. 2025 May 9;12:1562647. doi: 10.3389/fcvm.2025.1562647 (PMC12098648; doi:10.3389/fcvm.2025.1562647)
Supplement: Supplementary file 1 [file Table1.pdf]

Supplementary Material

Supplementary table 1. Baseline characteristics of patients by the presence of mild hyperkalemia at baseline.

**Characteristics**	Total Cohort<br><br>N=500	Serum K<5.0 mmol/L<br><br>N= 375 (75 %)	Serum K ≥5.0 mmol/L<br><br>N= 125 (25%)	P Value
Age (years)	58±13	57±13	60±12	0.03
Male 2	352 (70%)	266 (71%)	86 (69%)	0.65
BMI (kg/m^2)	26.8 (24.5 – 29.7)	26.9 (24.6 – 30.0)	26.3 (24.2 – 29.1)	0.076
LVEF (%)	27±8	27±8	28±8	0.57
Comorbid Conditions				
Hypertension	204 (41%)	149 (40%)	55 (44%)	0.40
Diabetes mellitus	225 (45%)	156 (42%)	69 (55%)	0.008
Ischemic heart disease	285 (57%)	206 (55%)	79 (63%)	0.11
Chronic kidney disease	145 (29.0%)	85 (23%)	60 (48%)	<0.001
Vital signs				
Systolic blood pressure (mmHg)	111 (100 – 125)	112 (100 – 126)	109 (100 – 120)	0.083
Heart rate (bpm)	74 (66 – 85)	75 (66 – 85)	74 (65 – 86)	0.50
Medications at baseline				
ACEi or ARB or ARNI	471 (94%)	351 (94%)	120 (96%)	0.32
Beta blocker	470 (94%)	349 (93%)	121 (97%)	0.13
Spironolactone	449 (90%)	340 (91%)	109 (87%)	0.27
SGLT2i	276 (55%)	207 (55%)	69 (55%)	>0.99
Laboratory values				
Serum sodium (mmol/L)	140 (138 – 142)	140 (138 – 142)	140 (138 – 142)	0.88
Serum potassium (mmol/L)	4.5 (4.2- 5.0)	4.4 (5.1 – 5.4)	5.2 (5.1 – 5.4)	---
Serum chloride (mmol/L)	104±4	103.6±3.8	104.4±3.8	0.085
Serum creatinine (mg/dL)	1.1 (0.9 – 1.3)	1.1 (0.9 – 1.3)	1.3 (1.1 – 1.6)	<0.001
Urea (mg/dL)	44 (34 – 58)	42 (32 – 53)	52 (41 – 76)	<0.001
eGFR (ml/min/1.73 m^2)	72±24	76±23	60±24	<0.001
NT-pro-B-type natriuretic peptide (pg/ml)	1496 (442 – 3460)	945 (310 – 2346)	1651 (541 – 3869)	0.065

Values are presented as n (%) or mean ± standard deviation unless specified.

BMI = body-mass index; LVEF = left ventricular ejection fraction; ACEi = angiotensin converting enzyme inhibitor; ARB = angiotensin receptor blocker; ARNI = angiotensin receptor – neprilysin inhibitor; eGFR = estimated glomerular filtration rate.
